# Perception of pregnant women towards early antenatal visit in Fiji: a qualitative study

**DOI:** 10.1186/s12884-022-04455-y

**Published:** 2022-02-10

**Authors:** Renita Maharaj, Masoud Mohammadnezhad

**Affiliations:** 1 Master in Public Health, Ba Mission Hospital, Ba, Fiji; 2 Associate Professor in Public Health, School of Public Health and Primary Care, Fiji National University, Suva, Fiji

**Keywords:** Antenatal care, Late booking, Perception, Pregnant mother, Fiji

## Abstract

**Background:**

Antenatal Care (ANC) is an opportunity to provide care to prevent potential maternal and new born mortality and morbidity and reduce new born mortality and morbidity. There has been an increase in the number of women receiving early ANC over the last two decades, however, in many developing regions such as Fiji, women are still delaying initiation of ANC. Therefore, the aim of this study is primarily to explore reasons for delayed initiation of ANC appointments and to explore knowledge and perception of pregnant mothers towards early antenatal appointments in Fiji.

**Methods:**

The study uses a qualitative approach. Data was collected among pregnant women more than 18 years of age after 12 weeks of gestation attending their first ANC clinic at the Ba Mission Hospital (BMH) from February 28 to April 2, 2020. Heterogenous purposeful sampling method was used to select 25 pregnant women for the study. A semi-structured open-ended questionnaire was used for face to face in-depth interviews. Data was analyzed manually using thematic content analysis after verbatim transcription of the interviews.

**Results:**

The mean age of the participants was 25.8 ± 5.9 years (age range of 19–40 years). The average gestational age of those making a booking for a consultation was 5.4 ± 1.4 months with a range of 4 to 8 months. The majority of women were multigravida (64%) and multiparous (40%). The main themes that emerged from the study were: i) perception of early ANC booking; ii) perceived barriers of early ANC booking and; iii) enabling factors of early ANC booking. Even though pregnant women have a good knowledge of when to initiate ANC, the practice of early booking was influenced by many other factors.

**Conclusions:**

The results of this study highlight the need to change the current booking system. Efforts are needed to attract the hard-to-reach women through outreach visits and increased communication between health care workers and the community with the use of community resources such as community health workers and traditional birth attendants. The media should be used to create awareness on timing and importance of early ANC visits at a community level.

## Background

Antenatal Care (ANC) is defined as the routine care of pregnant women provided between conception and the onset of labor [1] and is suggested as one of the pillars of safe motherhood by the World Health Organization (WHO) [2, 3]. ANC is an opportunity to provide care for prevention and management of existing and potential causes of maternal and new born mortality and morbidity [1, 4]. ANC often presents the first contact opportunity for a woman to connect with health services thus offering an entry point for integrated care. As a part of a global strategy to reduce the risk of maternal complications and stillbirths, the 2016 WHO ANC model recommends a minimum of eight antenatal contacts while advocating for a first ANC contact (also known as the booking visit) to be scheduled before the first 12 weeks of pregnancy [5, 8]. Two contacts are scheduled in the second trimester (20 and 26 weeks of gestation) and five contacts scheduled in the third trimester (30, 34, 36, 38 and 40 weeks) [5–7]. This replaces the previous four-visit Focused ANC (FANC) with the aim of improving the quality of ANC [6].

The new WHO ANC model (2002) for developing countries and the Royal College of Obstetrician and Gynecologists (RCOG) antenatal guideline (2003) recommended booking in the first trimester to afford patients the opportunity of early diagnosis of abnormalities and appropriate interventions [9]. Early ANC is defined as ANC before 12 weeks of gestation and is important for the best health outcome of the mother and the baby [2]. The goals of early ANC are to confirm pregnancy and the Expected Date of Delivery (EDD); classify women for basic ANC; to screen, treat and give preventive measures; to develop a birth and emergency plan and; to advice and counsel [8, 10, 11]. The estimated worldwide coverage of early ANC increased from 40.9% in 1990 to 58.6% in 2013. The overall coverage in developed countries was 84.8% while in developing countries it was 48.1% [1]. Many studies conducted in different developing countries show very low coverage of first ANC visit [12, 13]. Only 25% of pregnant mothers initiate ANC within the timeframe recommended by WHO [14].

Women who start ANC service early and continue regularly are likely to be delivered by skilled birth attendants compared to those who initiate ANC late and attend few ANC visits [15]. On the other hand, literature reveals that developed countries had a relatively lower prevalence of late booking [16, 17]. Approximations suggest that regions with low rates of early ANC coverage are the ones with high rates of maternal mortality [1]. Late booking contributes to still births and leads to high infant and maternal mortality rates [18]. Late booking means that women may not have the opportunity to benefit from screening tests, antenatal education and health advice, or supported decision-making regarding the place and choice of delivery [19]. Late booking will lead to missed early diagnosis and treatment of conditions such as gestational diabetes, fetal anomaly and intrauterine growth retardation, poor pregnancy outcome and an increase in total cost caring increases for a pregnant woman [20, 21]. Research has shown that maternal and neonatal death is avoidable with ANC being one of the key strategies [19, 22, 23]. In addition, initiation of early ANC is important as it is one of the components of achieving the Sustainable Development Goals (SDGs): 3.1 (Reduce Maternal Mortality Ratio (MMR) to less than 70 per 100,000 live births by 2030) and 3.2 (Reduce Neonatal Mortality Rate to less than 12 per 1000 live births and U5MR (under five mortality rate) to less than 25 per 1000 live births) [24].

The MMR for Fiji that have gradually fallen from 49 deaths per 100,000 live births in 1996 to30 deaths per 100,000 live births in 2015 [25]. WHO Reproductive Health Publication demonstrates there has been a consistent reduction in maternal mortality over the years. The rate of improvement has slowed since 2010 which suggests a further marked reduction in MMR would require additional efforts by government, clinical service networks and community and development partners [26]. Even though ANC coverage in Fiji has reached more than 95% with many pregnant women achieving more than four visits, less than 10% of women are booked in the first trimester [27]. Since 2012, there is higher coverage for women who had four or more visits during pregnancy than their first antenatal visit [28] yet little is known about the factors contributing to late initiation of ANC in Fiji.

Women who have a good perception of early ANC visit are more likely to initiate ANC earlier than their counterparts [12, 29]. No study has been conducted in Fiji or the Pacific to determine the prevalence or the factors delaying initiation of ANC. The current study is the first of its kind in Fiji and in the Pacific that will address the knowledge gap and build on existing literature by looking at issues that are still unclear in relation to factors which delay early booking by pregnant mothers. The findings from the study can assist in planning local programs and interventions to ensure pregnant women has assistance to initiate ANC during the first trimester of pregnancy to receive optimal care. Finally, the current study can help guide future local studies and encourage more studies to be conducted in Pacific Island Countries (PICs) to gain a better understanding on why mothers delay antenatal booking.

## Methodology

### Study setting and design

This was a qualitative study conducted in the Ba Mission Hospital (BMH) from February 25 to April 2, 2020. The BMH is run by the Ministry of Health and Medical Services (MoHMSs) and is the sub-divisional hospital of the Ba Sub-division that provides a range of services free of charge to its population of about 55,000. The population comprise of i-Taukei and Fijians of Indian descent, the latter making-up the largest group. The main languages spoken are Fijian and Hindi. The majority of people use the public health facilities. ANC booking is done every Thursday from 8am to 12mid-day. The number of patients booked range from 15–30.

### Study population and sampling

The population of the study was all pregnant women attending booking antenatal clinic after 12 weeks of gestation in the BMH within the study period. There was a total of 61 women who came for booking during the study period. The sample was chosen through means of heterogeneous purposive sampling. Women, more than 18 years of age from Ba and who were attending their first ANC clinic in BMH after 12 weeks of gestation were included in the study. Women who were not willing to participate in the study, those who were either booked or resided outside Ba sub-division, women who were mentally and physically incapable of being interviewed, or those who need urgent medical attention or are in labour at first booking were excluded from the study.

The idea of this sampling was to look at subjects from all available angles to gain a better understanding of participants and to involve candidates across a broad spectrum relating to the topic of the study. There was age variation in the recruitment of participants to ensure the capturing of opinions of across the age range. Participants were also selected based on their gravidity because women experiencing pregnancy for the first time will have a different perception of ANC than a woman who has experienced it more than once. Since marital status has an influence on the timing of initiation of ANC, it was ensured that both married and unmarried women were interviewed. Those who were either booked or lived outside Ba and were mentally and physically incapable of being interviewed, or those who needed urgent medical attention or were in labor at first booking were not included in the study. Eligible women were given information and were asked for voluntary written consent. The study aimed to include a variety of participants and interviews continued until no new themes emerged. A total of 25 pregnant women were interviewed during the study period.

### Data collection tool

Face to face in-depth interviews were conducted with the participants through the use of semi-structured interview guides. The design of the questionnaire was informed by insights from exploratory studies and existing literature. The study focused on three areas: (i) demographic characteristics; (ii) 6 main/core questions that explored on knowledge, perception, barriers and facilitators of antenatal booking; (iii) probe and follow-up questions.

### Study procedures

The sister in-charge of the antenatal clinic was informed about the study verbally and flyers were posted in the antenatal waiting area to advertise the study. While mothers were waiting to be booked for ANC, a verbal introduction about the study was done with the help of a translator. The information sheet was then provided to the mothers in three different languages; English, i-Taukei and Hindi for them to read and decide if they wished to participate or not. Participation in the study was voluntary and did not inhibit the respondents’ access to care. Those who were willing to participate met the researcher after their booking clinic in the antenatal waiting area. The eligible women interested in participating in the study gave voluntary written consent before they participated in the in-depth face to face interview which lasted for 25–35 min. Most of the interviews were conducted either in English or Hindi. For those who couldn’t speak Hindi or English, a bilingual translator was engaged. Privacy was maintained by conducting the interviews in an isolated room with no-one else around. Participants were permitted to only discuss issues with which they felt comfortable. The researcher asked questions relevant to antenatal booking. Consent was also obtained from a bilingual translator to assist the researcher in translating i-Taukei and a statement of confidentiality was signed by the translator as well. Data was recorded through audio taping and notes was taken to capture non-verbal cues and biographical information.

### Data management

Notes taken during the interview was validated during transcription by listening to tape recordings that were made during discussion sessions. Summary transcriptions of hand-written notes from the interview were also prepared every day immediately after collection and they were elaborated on later after completing the entire study. Immediately after each interview, audio recorded interviews were listened to for any clarification and to know if there was any need for follow-up interviews. Each audio-recorded interview was transcribed verbatim and analyzed one at a time. After transcription the data was checked for accuracy and sufficiency. The transcript was manually analyzed for data analysis using Thematic Content Analysis (TCA). The following process involved familiarization, coding, identifying themes, reviewing and refining, integration and interpretation [30]. Each transcript was read and re-read multiple times to become familiar with the data in order to develop a deep understanding of its contents. The textual data was disaggregated to examine the similarities and differences in the data while grouping similar data. Similar data was coded. This involved assigning a topic to the paragraph relating to the issue of interest in the study. A list of codes was developed with a brief description of each. New codes were assigned to passages that could not be coded with previous codes. Connections across codes were searched for to develop themes. New themes were created as the list of codes emerged. After reflection, their commonality was identified and a broad descriptor was assigned to them. Some themes became sub-themes of others. Finally, a master table was constructed containing themes, sub-themes and quotes from the transcript.

### Study rigor

The researcher adhered to criteria of ensuring trustworthiness to ensure rigor as described by Polit and Beck, (2012): credibility; dependability; conformability and; transferability [31].

*Credibility* was ensured through prolonged engagement with the participants; member checking where participant’s response were taken back to them for their view point or alternative interpretation; triangulation, was ensured through audio recording and field notes of both the interviews and peer debriefing whereby the study process was continuously discussed with the supervisor. To ensure *dependability,* all interviews had verbatim of transcription, direct quotation and inclusion and exclusion criteria will be used. *Transferability* was ensured through the use of purposive sampling, description of research methods and data saturation. The methodology is thoroughly explained for anyone who wishes to repeat the study. Tape recording and field notes will ensure *conformability*. A description of the participants and the use of verbatim transcription ensured authenticity*.* In addition, all signed consent form will be safely kept in case of evidence.

### Ethical considerations

The ethical clearance to conduct the study was obtained from the College Health Research Ethics Committee (CHREC) at Fiji National University (FNU) and Fiji National Research Ethic Review Committee (FNRERC) and approval from Sub-divisional medical officer (SDMO) of BMH was granted before the commencement of this study. Informed Consent was provided by all the participants.

## Results

### Characteristics of the participants

There were 25 pregnant women who participated in in-depth face to face interviews. They were all booked after 12 weeks of gestation. The mean age of the participants was 25.8 ± 5.9 years (age range of 19–40 years). The average gestational age of booking was 21.6 ± 2.6 weeks with a range of 16 to 32 weeks. The majority (64%) of participants were i-Taukei, married (56%), unemployed (88%) and almost all were from rural areas (96%). Their education level differed from primary to secondary to tertiary with secondary level education comprising the majority (72%). The majority of women were multigravida (64%) and multiparous (40%); the gravidity of multigravidas ranged from 2 to 5 while the parity of multiparous ranged from 2 to 4 (Table 1).

**Table 1:** Characteristics of the pregnant women (*n* = 25)

Characteristic	Frequency (Percentage)
Age (Mean ± SD)	25.8 ± 5.9 yrs
18–34	22 (88)
> 34 years	3 (12)
Ethnicity	
i-Taukei (IT)	16 (64)
Fijian of Indian Descent (FI)	9 (36)
Marital status	
Married	14 (56)
Single	11 (44)
Employment	
Employed	3(12)
Domestic Duties	20 (80)
Student	2 (8)
Residence	
Urban	1 (4)
Rural	24 (96)
Education Level	
Primary	3 (12)
Secondary	18 (72)
Tertiary	4 (16)
Gravidity (G)	
Primi gravida (1 pregnancy)	9 (36)
Multigravida (2 – 5 pregnancies)	16 (64)
Parity (*P*)	
Nulliparous (0 previous live births-PLB*)	9 (36)
Primiparous (1 PLB)	6 (24)
Multiparous (2-4PLB)	10 (40)
Gestational age at booking (weeks)	
Mean ± SD	21.6 ± 2.6

^a^PLB – previous life birth

### Thematic analysis

After conducting a manual thematic content analysis, 3 major themes were identified; perception of early ANC booking; perceived barriers to early ANC booking; and enabling factors for ANC booking (Table 2). In this section, G and P imply gravid and parity of the pregnant women respectively. For instance, G4P3 means 4 pregnancies and 3 previous live births. IT indicates i-Taukei and FI indicates Fijian of Indian descent.

**Table 2:** Themes and sub-themes of in-depth interview with the patients

Themes	Sub-themes	Codes
1. Perception of early ANC booking	i) Knowledge of ideal timing of booking	Correct knowledge, incorrect knowledge, no knowledge
	ii) Importance of early ANC booking	Detection of medical problem, for investigation purpose, for education and counselling
2. Perceived barriers of early ANC booking	i) Socio-demographic factors	Financial constraints, geographic location, mode of transport, changing residence, employment status
	ii) Socio-cultural factors	Unplanned/unwanted pregnancy, ignorance of sexual reproductive health, inadequate knowledge
	iii) Obstetric factors	Delayed diagnosis of pregnancy, child spacing, multigravida, past pregnancy experience
	iv) Health care system factors	Attitude of the health care workers (HCWs), lack of required documents, limited opening hours, long waiting time, lack of advice from HCWs
3. Enabling factors	i) Family resources	Advice from others, partner support
	ii) Community Engagement	Traditional Birth Attendants (TBA), church leaders
	iii) Health care service related factors	Adequate advice from health staff on timing of booking, increased awareness on requirements for booking, flexible opening hours for the clinic, booking by outreach team, kid-friendly ANC, positive relationship between the HCW and the pregnant woman

### Theme 1: perception of early ANC booking

When talking about ANC, the common topics the pregnant mothers raised were knowledge on ideal timing for booking, value of early ANC and knowledge of mothers about benefits of attending ANC early.

#### Knowledge on ideal timing for booking

It was found that most women had correct knowledge on time of starting ANC. Their understanding was that it is important to start booking within the first trimester so as to enable them to receive the care needed. Their perception of early booking ranged from one to six months. 7 participants said that booking should take place within 1 month of pregnancy; 5 participants said booking should be done within 2 months, 7 participants said within 3 months. 6 participants, however, did not have the knowledge about when to start ANC; they said that ANC booking should start after he the first trimester.

“…*not sure at which age to book…maybe before due date…’ (*20yr ITG1P0)

*“…maybe two or three months…”* (26yr IT G4P3)

*“…booking should be done at 6 months…”* (39yr IT G5P4*“...I really don’t know early or late booking…”* (25yr old ITG4P3)

Having the relevant information on timely booking and importance of early booking might assist mothers to book early. Therefore, one of the deterring factors to early antenatal booking in the current study was lack of knowledge on timely booking and importance of early initiation of ANC.

#### Importance of early ANC booking

Overall, there was a positive perception among woman towards starting ANC early. The study discovered that women understood the importance of starting ANC early and receiving treatment, especially if any problem was diagnosed. Some of the responses were:

“…*Early booking is important so that we have any problem it can be picked up earlier and we can work towards that problem…”* (40yr FI G4P3)

*“...early booking is very important because it will help us detect sickness and improve mind of the mother*...” (40yr old FI G4P3)

*“...Early booking means problem detected and managed early. Late booking means if any problem then nothing can be done…”* (24yr old FI G1P0)

Participants portrayed that early booking was important to assess the blood level of the mothers, to detect pregnancy complications earlier and to receive the right advice. Women also understood early booking meant an early scan.

“…*early is better, would have known my condition. Like today my blood is low…now unnecessary hassle…Late is today what I am doing...” (*19yr FI G1P0)

*“…early booking means we have scan done…that way we know if any problem with the baby early…if come late then it’s too late to know problem with baby…”* (39yr IT G5P4)

Participants expressed it was better to come early for booking so they do not miss out on education and counselling sessions which occurs in early pregnancy.

*“…Early booking is better because some of the new mothers do not know a lot…if they come late they will miss the education and advice that we get on booking…like this way we know what things to do but if we come late then we miss onto these things…”* (36yr old FI G5P4)

### Theme 2: perceived barriers to ANC booking

The study found that most women are not starting ANC early because of the perceived barriers. Some of the barriers identified in the current study were socio-demographic barriers, socio-cultural barriers, obstetric and health service-related factors.

#### Socio-demographic factors

The study showed that financial problems were one of the barriers to initiating timely ANC. Women from poor families living far from the health facilities could not afford transport costs. Few participants reported distance to heath facility which was worsened by lack of transport that contributed to their late booking. Furthermore, some pregnant women were traveling by public transport which came at scheduled times and it did not match with the booking time.

“…*because before I stay very interior in Ba, have to pass 4 village and hospital is in the fifth village*…*wanted to come early but hospital is very far, transport is too difficult like $10 fare…can come on horse ride but too risky…”* (25yr old IT G1P0)

One participant had said that the unfavorable weather conditions together with transport problems delayed her timely initiation of ANC.

*“…Doctor I went to ANC booking clinic and told them that I was late as there was flooding in the area where I lived and I had no transport to get me across for booking…”* (23yr old FI G2P1)

The findings of the study revealed that participants changing residence and family problems were also a hindrance to early booking.

*“… Because of family problem I was shifting here and there…wasn’t sure of where I’ll be staying…I was in Ba then temporarily staying in Lautoka, husband just got transferred to Ba so I came to be booked here…”* (19yr old FI G1P0)

Moreover, it was revealed from the study that employment status of the pregnant woman influenced a woman’s decision on the time of antenatal booking.

*“…u know doctor I am a working mother, it’s not easy for me to come for booking, we are short of staff…I would have come but if I get kicked out of my job then who is going to look after my family…no one will cover my shift…so I come when I have time to come for booking…”* (30yr old IT G4P3)

#### Socio-cultural factors

The study revealed that some women postpone timely ANC booking because they initially didn’t want the pregnancy and they only came for booking when they decided to continue with the pregnancy. Unplanned and unwanted pregnancy eventuated to passive acceptance. This is evident from the following remarks of the participants.

*“…I didn’t intend to have the baby, I was on family planning, I just missed my menses once and I get pregnant…”* (30yr old IT G5P5)

It was also discovered that the participants were not prioritizing pregnancy as a few women intended to seek antenatal care at a time convenient to them. ANC booking wasn’t given much importance by them and they engaged in other activities because pregnancy was a natural process. Women had other commitments which were more important to them than having booking on time.

*“…should come in February but have some occasion in Suva…important family occasion doc couldn’t have missed…wanted husband to come with me but he was at work all the time…today he couldn’t come either but I had no choice as I am already late for booking…”* (20yr G1P0 IT booked at 4 months)

*” ...I was like very busy at home so no time to come…”* (26yr IT G4P3 booked at 5months)

Fear of consequence of disclosure of pregnancy also influenced a women’s ability to seek timely antenatal care in the current study. One woman postponed her antenatal care until the time she disclosed the pregnancy to her parents.

*“…i was afraid to inform my parents about the pregnancy, just like a week ago they were informed and then I came for booking”* (19yr old IT G1P0)

Some women had the wrong perception about the timing and purpose of early initiation of ANC. Wrong perception can be related to lack of knowledge of ANC as well as cultural and traditional beliefs related to health-seeking behaviour during pregnancy. A 24-year-old i-Taukei G2P1 woman had her pregnancy confirmed at 2 months but because there was no visible sign of pregnancy she didn’t come for booking. However, she resorted to another lady in her village that massaged her stomach and told her she was not pregnant. She again resorted to a pastor when she started feeling the movement of the baby. The pastor had to re-confirm her pregnancy before she came for booking at 5 months. Another 23-year-old IT G2P1 went to a Traditional Birth Attendant (TBAs) to get her stomach massaged and confirm her pregnancy despite the nurse’s advice.

*“…I went to Namau nursing station and was told by the nurse to come for booking…not sure pregnant or not first 3 months so I went to one lady who delivers baby. She massaged my stomach and told me I am pregnant then I went to Namau nursing station…”* (23yr IT G2P1)

Some of the pregnant women had the perception that the main aim of the early booking was to know about the state of the baby. If the baby wasn’t fully formed, then early initiation of ANC care was a waste of time. Another 23-year-old FI female discussed how it was all right to book late as it was her individual perception that an early scan would not show a viable foetus.

“…*Pregnancy diagnosed at 4 weeks but scan was just showing a dot, came only when baby was seen fully on scan…”* (23yr FI G2P1)

#### Obstetric factors

Delayed diagnosis of pregnancy was another cited factor that postponed booking. Many women interviewed said that they were not aware of being pregnant and because pregnancy was diagnosed late, they came late for ANC booking.

Some women reported having the “normal” cycle irregularity which delayed their access to early antenatal care while a few did not notice signs such as amenorrhea as the cardinal sign of pregnancy.

“…*unsure initially if pregnant or not…I always miss my period for two to four months but this time more than 5 months…”* (27yr FI G3P2)

Moreover, some pregnant women recognized their pregnancy late because of conceiving while lactating or continued to have menstruation even after conception.

*“…no signs of pregnancy unlike with the other kids…i am breastfeeding so unsure as wasn’t having any menses…only last week Friday pregnancy test was done…”* (22yr old IT G2P1)

One woman had attributed her delay on timely booking to failure of family planning. Therefore, lack of knowledge about the chances of getting pregnant while on family planning and the time frame for the side-effects of family planning medicine to disappear delayed accessing ANC service.

*“…on family planning doc, was on Jadelle which just got removed. So, didn’t expect to be pregnant…”* (25yr old G5P4)

Child spacing was another barrier for multiparous pregnant women to timely initiation of ANC. Participants who had younger children reported that they had to plan for children before they could come for booking.

” *…my two kids going to school. Have to look after the youngest one who is 2 years of age…today I arrange my sister to look after him so I came…”* (26yr old IT G4P3)

It was also highlighted in the study that multigravidity can be one of the barriers for early ANC initiation. Women who have delivered before think that they need not come for early booking. Therefore, it is the perception of these multigravida women which makes them come late for booking.

*“…I have delivered before and I know what all has to be done during pregnancy…”* (30yr old It G4P3)

The study also revealed that experience of previous pregnancy influenced timing of ANC booking. A good past experience of pregnancy influences the decision of few women to postpone their booking. Late booking for some also meant less antenatal care visits.

*“…this is my fourth baby so I know what to do, I can handle it, the food I eat and everything…late booking means less hospital visits…”* (30yr old It G4P3)

#### Health care system factors

Poor attitude of the health care workers towards patients had an effect on when to start ANC. Respondents cited how they anticipated the attitude of the HCWs towards them will depend on their relationship status. They discussed HCWs posing a lot of questions if women were in unstable relationships and this was portrayed by the following statement:

*“… went to CWMH initially but some senior staff were judgemental, they would look at me knowing I am not married and then I know they will ask me all sorts of questions only in the end to tell me to go away if I don’t have the marriage certificate …”* (24yr. old FI G1P0)

A multiparous shared how her previous encounter with the HCWs affected her current pregnancy and booking. She had the following to say:

*“…for me I am just scared of hospital. I have delivered before and have gone through the process and just the thought of it makes me so scared. I am scared of the nurses who book, if I come late they will be angry...like today I forgot to bring my urine and they were angry…”* (30yr old IT G5P4).

Another respondent expressed how she presented multiple times to hospital for vomiting but none of the HCWs had told her about booking. She said they would just treat her vomiting and she would be admonished for presenting at the hospital several times for vomiting.

*“…I came for vomiting to hospital, only tablet given, no advice on booking. They treat my vomiting and send me away, they think I come to hospital unnecessary and want to be admitted. I get scolded at but no one talks about booking…”* (27yr old FI G3P2)

Single participants or those who were in unstable relationships along with an inability to produce the required documents was seen as another factor that contributed to delays in antenatal booking. A few participants in the study also expressed that the lack of a marriage certificate was the reason for their delayed initiation of ANC. Some of the participants were not legally married and marriage certificate was a pre-requirement of booking.

*“…I am my husband’s second wife. Husband not yet divorced but now they are but it will take 21 days for the marriage certificate to be done…all kids booked at 3months if no legal document required then would have come earlier…”* (26yr old IT G4P3)

The current study also showed that the schedule and timing of ANC booking in BMH had an effect on timely initiation of ANC. According to some participants, they had delayed their booking because they would not be allowed to be booked if they came late even if the clinic was open.

*“ …my intention was to book on time doctor but Because of flooding I reached clinic after 11, the clinic was still open and I was told that it was too late so I had to go back…I had already used the money for the transport and had to wait for next month…”* (23yr old FI G2P1)

A few of the participants were still schooling and they reported the clinic hours as the main barrier for them not to book on time.

*“…doc I am still schooling…booking will mean I have to miss school because I have classes on Thursdays and my classes are almost always same time as booking…”* (19yrs old IT G1P0).

Long waiting time with overburdened health facilities were also reported as barriers to seeking early ANC. The following responses attest to that:

*“…also booking is only 1 day in a week and from 8am to 11am. It is difficult to reach on time, you know doctor there is so many things to be done…went to records people were so crowded, don’t know who gets the priority so waited and waited and by the time I went for my booking it was so late and I was really tired…”* (20yr old It G1P0)

Insufficient or lack of proper advice on booking from the HCWs was reported as reasons for delayed booking by some of the participants. When asked about the source of information about ANC services, the majority of the women mentioned they had never received any advice about ANC booking from the health staff, which was very surprising. Even if they did receive advice on booking from a HCW, their instructions were not clear enough for them to initiate booking on time.

“…*the doctor had advised me on early booking…never tell me what all to bring only booking time…” (24yr old IT G2P1)*

### Theme 3: enabling factors

Enabling factors are conditions or factors that make ANC booking available for the women. The study highlighted having factors such as good family support, having effective TBAs and provision of good quality health services enabled the women to start ANC booking at the right time.

#### Family as a resource

Respondents who made first ANC contacts did so because they were encouraged by their husbands or close family member to seek care. Encouragement and support from relatives and other women previously pregnant provided positive influence with ANC boking. The majority of participants acknowledged that they were motivated by their husbands to come for booking, 4 respondents were encouraged from their mother-in-law and/or sister-in-law), 2 said their neighbours had encouraged and advised them on booking. Therefore, the support from the family was more social than financial in nature that enabled women to come initiate ANC early.

*“…my husband advised me to book at 21/2 months but I was scared they will ask for the marriage certificate…”* (40yr old FI G4P3)

*“…my family motivated me to come for booking…my sister is a nurse so she advised me…”* (19yr old FI G1P0)

A husband’s support included accompanying his wife for the booking clinic; encouraging her to attend the clinic; providing bus fare to attend; and taking care of their other children. The family also played a supportive role such as looking after the children, helping with domestic duties.

*“...now my sister is at home looking after the kids, she came early so I can come for booking…*” (25yr old IT G4P3)

#### Community resources

From the study, is was shown that the community enabling factors such as the availability of Traditional Birth Attendants (TBAs) and pastors were factors that enabled the mothers to attend ANC booking. The following is one of the responses:

*“…not sure pregnant or not first 3months so I went to one lady who delivers baby. She massaged my stomach and told me I am pregnant then I went to Namau nursing station…”* (23yr old IT G2P1)

#### Health care services and HCWs

The provision of free medical services and food vouchers for mothers with first three children enabled women to come for booking.

The message which is conveyed by the HCW about when to attend ANC seemed to influence the timing of booking in the current study. Respondents cited that the advice the doctors or the nurses gives should be very clear in terms of the opening hours of the clinic and the required documentation to bring along to booking.

*“…doctor should tell us what day the booking is in detail and the requirements once pregnancy is confirmed…new mothers do not know the requirements, the first doctor who sees us should tell us about the requirements…”* (23yr old FI G2P1)

Some of the respondents also expressed that there should be printed notices about the ANC booking requirements.

*“…doctor should tell us what day the booking is in detail and the requirements once pregnancy is confirmed…new mothers do not know the requirements, the first doctor who sees us should tell us about the requirements…”* (23yr old FI G2P1)

A few respondents cited if there could be more flexibility with booking times. One participant recommended that booking should be done by the nurses during home visits or outreaches for women who were having problems accessing the health services.

*“…we live very far, if the nurses can come visit us, come check, blood test…”* (25yr old IT G1P0)

A 27 yr old FI multiparous respondent suggested if the ANC clinic can be more supportive in terms of accommodating children when a woman attends booking.

The health care provider–client relationship has an effect on the care given to the women. The latter tend to understand information and appreciate the care given to them when they are treated well by the nurses. They tend to feel at ease when they are treated in a positive manner. The majority of the participants were appreciative of the advice and counselling that was provided to them and were overall satisfied with the booking service.

*“…first of all, I was scared that I will get the growling from the nurse but when I saw their smiley faces I was happy and their words of encouragement I knew I was at the right place. i received a lot of advice and support today…”* (30 yr old IT G5P5).

## Discussion

The aim of this study was to explore reasons for delayed initiation of ANC appointments and to explore knowledge and perception of pregnant mothers towards early antenatal appointments in Fiji. After doing manual thematic content analysis, 3 major themes were identified: perception of early ANC booking; perceived barriers to ANC booking; and enabling factors for ANC booking. Factors such as an individual’s socio-demographic characteristic; their awareness of existing services; their perception of those services; along with their cultural norms; disease patterns; and local health system service influence health seeking behavior [32].

Therefore, some of the ways to increase first antenatal care visits in this setting would be through the provision of health education for the women on the importance of early ANC booking using mass media such as radio, television as well as through health talks during outreach visits by CHWs. It is important to train the CHWs to identify pregnant women in the community and counsel them on the need for early ANC booking and care by doctor and nurses at birth. Because CHWs live in the community, they have a better understanding of local lifestyles and beliefs which might affect a woman’s knowledge and attitude towards ANC. CHWs create a link between the community and the health care system while reinforcing health messages. Pregnant women with adequate knowledge on the importance of early ANC booking will result in good attitudes and practice among them [32] Education and awareness of early signs of pregnancy can improve diagnosis of pregnancy and hence earlier booking [10]

Physical inaccessibility has been shown to be a significant barrier for adequate ANC coverage. Some of the respondents included physical inaccessibility, where a nursing station was a considerable distance from where they lived. For rural areas with no health care facility, factors such as distance from the health facility and mode of transportation may also contribute to late initiation of ANC [4, 10, 22]. Hence, formal decentralization of ANC services to the nursing station as well as conducting mobile clinics for hard to reach areas might improve early uptake of ANC by decreasing the distance a woman has to travel to access ANC. It is not just the physical infrastructure that matter when it comes to maternal health [33] but also the availability of transport and affordability of use as was evidenced in the study. Health service is complemented by transport and road. Mode of transport used by the women affected the timing of initiation of ANC in the current study, for instance, women who were using public transport had to catch the very first bus for them to make it on time for the ANC otherwise if they come later they will be asked to return only to come back at a later date.

It was shown that cultural practice and traditional beliefs played a part in health seeking behavior of the women in the study setting. It was established from the study that i-Taukei women did not start ANC on time as they waited for their church pastors, TBA or mother-in-law to provide advice first. This leads to pregnancy being concealed until the pregnancy is 3—4 months. This was also noted in studies conducted in Malawai and the Republic of Congo that showed culture and religion influence with ANC attendance and the practices attached to these beliefs delay initiation of ANC [13, 14]. Mathole, et al., (2004) proposed that women who are highly religious might trust their ethnocultural and religious practice in ensuring good health during pregnancy thereby presenting late to health care facility for ANC [35]. Thus, it is vital to be aware that health beliefs regarding pregnancy still exist in Fiji which can increase the complacency in health seeking practices by women. HCWs need to be mindful of the traditional beliefs and practices in order to develop appropriate messaging tools regarding early ANC that encourage positive beliefs and practices while discouraging the negative ones. Trained focal persons such as TBAs, religious leaders, and other opinion leaders working as community volunteers in close collaboration with existing community structures and health services have been found to be effective promoters of obstetric care but also of early and frequent utilization of ANC in Southern Tanzania [36].

In the current study there were some unplanned, and unwanted pregnancy which might have led to ignorance of amenorrhea and consequently to book late. Studies have shown unwanted pregnancy [37, 38] and abortion [39] are related to late booking. Mkandwire. P, et al., (2019) concluded that women with unintended pregnancies are more likely to delay ANC than women with intended pregnancies [14]. Sinyange, et al., (2016) revealed that women who did not want to have any more children had an increased chance to delay ANC booking [40]. Women delayed confirmation of pregnancy or delayed booking if they were not ready for another child. Unplanned pregnancy could also be related to the initial denial of the pregnancies themselves and concealment from others. Consequently, women are less motivated to attend early ANC booking. This could be because they have a fear of the response and judgement of father or relatives, unable to cope with another child and failed termination of pregnancy. There was an effect on booking when a pregnancy was unplanned and unwanted especially if the woman was unmarried and/or young [41]. This warrants the need for family planning services in communities to be readily available and easily accessible. The knowledge of women on contraception has to be increased such as the failure rates of each contraception as it was revealed in the current study that women were delaying their ANC visit because they were on contraception. Raising awareness on family planning will also contribute to a woman’s knowledge on reproductive health and will improve utilization of ANC service. The findings from the current study suggests that pregnancy test kits should be available in the health facility for earlier diagnosis of pregnancy especially for women who cannot afford to buy the kit who then wait for scan to confirm pregnancy.

Multiparity was one of the factors contributing to delayed initiation of ANC in the current study. These findings were similar to those in other studies that showed that multiparity was a significant determinant of late booking [42]. For primiparous women, the diagnosis of pregnancy and the joy of living this new experience for the first time might have influenced the decision of these women to seek medical care earlier in their pregnancy. Multiparous women might be more relaxed about early ANC booking because of their previous positive pregnancy experience and outcomes and their beliefs that there was more to be learnt with first pregnancy. This could also mean that women are inadequately educated in ANC about the importance of presenting on time for subsequent pregnancies. They feel starting early is not necessary if they had no complications during the previous pregnancy. Multiparous women might also be finding it difficult to look after other children as was highlighted in the current study.

Moreover, ANC attendance is responsive to the attitude of the health staff; positive or negative impact on the ANC turnout to the standard of WHO recommendations. It was also highlighted in the present study how it was difficult for women to build rapport with the HCWs as they were unapproachable at times. Efforts should therefore be made to provide respectful care and eliminate disrespect and abuse in health care settings to enhance ANC uptake and attendance. Women tend to feel at ease when they are treated in a positive manner. When women are provided with information regarding ANC, pregnancy risk and danger signs, they are more motivated to initiate ANC early to avoid risks associated with pregnancy. Health staff who provide ANC exercise significant authority and women generally place trust in the instructions they give. Therefore, messages about when to attend ANC communicated by health staff seemingly influence ANC attendance. Ambiguous instructions can result in delays to ANC. There is a need to strengthen the communication strategy from the service-providers side. Improving the perceived quality of care given to women can improve early ANC booking, Notices should be put-up in hospitals to explain the opening hours of the ANC and the necessary documents required to avoid turning the patients away on the day of booking. IEC materials in different language should be available in the waiting areas of the hospital to raise awareness of early ANC booking. Before a woman comes for her first ANC, she needs to understand it can be a whole day procedure and subsequently will come prepared for long waiting hours.

Furthermore, the need for the marriage certificate for first ANC visits serves no other function other than for validation of data and therefore should not be a requirement for booking. omen should not be told to return at a later date for failure to show a marriage certificate. Women should not only be booked at the fixed scheduled ANC clinics of hospitals but should be booked at the first encounter with the health care setting, be it be a nursing station, health center or outpatient department of any sub-divisional hospital with a nurse, nurse practitioner or a medical officer. Reaching out to women who are unable to access care through outreach visits is another way in which barriers to access can be reduced. Multi-disciplinary approaches with the involvement of the CHW, zone nurse and the zone doctor will assist in early initiation of ANC by the HCWs.

As with other studies, the current study demonstrated that even though early initiation of ANC was important, it was something that wasn’t given immediate priority and could be procrastinated by women. few women knew that they were pregnant but yet they chose to attend their ANC booking at a time convenient to them. The beliefs about the importance of ANC are not always predictive of behavior and do not account significantly for lack of use [39]. Most women accept the importance of ANC “in theory”. For it to be acceptable” in practice” it needs to be appropriate and a ‘good fit’ to the woman [39]. Lilungulu et al., (2016) in Tanzania showed that out of the 74.2% of the interviewed woman who had agreed on the importance of early booking, only 12.4% came for early booking [43]. An American study of black women of low socio-economic status found that late bookers viewed ANC as important, but not as important as other issues that they faced on a daily basis [38].

However, the study revealed also revealed enabling factors for timely initiation of ANC such as the woman having a supportive family. Partner involvement in terms of accompanying the women for ANC or initiating or supporting early use of ANC may have an important impact on early ANC visit. Studies have shown that the involvement of husband to book for ANC seemed decisive [10, 34]. Thus, Fiji should have a policy in place that promotes male involvement in ANC so that women are accompanied by their male partners. Women’s autonomy is also a significant predictor of delayed initiation of ANC, where non-autonomous women are more likely to delay ANC booking. This could be because they strictly have to comply with family norms; they are under the influence of their partner or family (parents-in-laws, particularly); they lack family or social support; or simply because of the refusal of the partner to accompany the women to the ANC booking. Hence, the ANC booking should be made “male friendly “to increase male participation. Policies should be reviewed in order to increase male involvement in maternal and child health care. Targeting mothers-in-law and husbands in health promotion and educational intervention could be a promising strategy to improve early initiation of ANC services in Ba.

There is a need for more research at a national level to determine the prevalence of first trimester booking and to look at factors that affect initiation of ANC through mixed method study for evidence-based policy development along with strengthening of existing policies to improve timely booking of ANC.

### Limitation of the study

Participants were not randomly selected and only those attending ANC clinic were chosen, therefore the results are neither representative nor generalizable. Only pregnant women were interviewed; Women who were booked in other health centers or with private General Practitioners (GPs) were not included in the study. The study reported views of pregnant women who attended ANC in BMH. Furthermore, the research did not include adolescent pregnancies who are likely to book late for their ANC visits, and women who were unbooked were not interviewed, thus their reasons for not having any ANC visit was not explored. Language and cultural differences between the researcher and the respondents might have inhibited the response of the participants.

## Conclusion

The study revealed that pregnant women are prevented from initiating timely ANC visits due to barriers which range from socio-cultural to health care service factors despite the women having a positive perception towards early antenatal booking and free provision of services.

Understanding these factors should therefore inform policy makers on how to influence women to attend ANC bookings in a timely manner. The key areas identified affecting ANC initiation should be targeted for during interventions. Any intervention should be culturally appropriate and potential interventions should focus on improving the attitudes of the wider public towards care; not the individual woman. There is a need to take antenatal services nearer to women through outreach programs which will aid in removing the location disparities. Outreach clinics need to be conducted by qualified staff such as a public health mid-wives.

Moreover, the information and counselling needs of women should be addressed. Awareness on early antenatal bookings should be conducted through the use of mass media such as television or radio. Community-based information, health education and communication on ANC services are important to overcome women’s perception of late booking. The booking process should be reviewed while paying attention to the reported barriers such as making the opening hours of the ANC more flexible.

## Data Availability

The datasets generated and analysed during the current study are available in the Open Science Framework (OSF) repository, https://doi.org/10.17605/OSF.IO/3YME9.

## Abbreviations

ANC: Antenatal Care
BMH: Ba Mission Hospital
CHREC: College Health Research Ethics Committee
EDD: Expected Date of Delivery
FNRERC: Fiji National Research Ethic Review Committee
FANC: Focused Antenatal Care
CWMH: Colonial War Memorial Hospital
GPs: General Practitioners
HCWs: Health Care Workers
MMR: Maternal Mortality Ratio
MoHMSs: Ministry of Health and Medical Services
SDGs: Sustainable Development Goals
SDMO: Sub-divisional medical officer
TCA: Thematic Content Analysis
WHO: World Health Organization
